# Genomic population structures of microbial pathogens

**DOI:** 10.1098/rstb.2021.0230

**Published:** 2022-10-10

**Authors:** Kathryn E. Holt, David M. Aanensen, Mark Achtman

**Affiliations:** ^1^ Department of Infection Biology, London School of Hygiene and Tropical Medicine, London WC1E 7HT, UK; ^2^ Big Data Institute, Oxford University, Oxford, Oxfordshire, UK; ^3^ Warwick Medical School, University of Warwick, Coventry CV4 7AL, UK

## 1. Introduction

A Royal Society Hooke Scientific Discussion Meeting to address ‘Genomic population structures of microbial pathogens' was originally scheduled for the end of March 2020. That discussion meeting was conceived when a ‘big-data’ problem became evident once more than 100 000 genomes of *Salmonella* had been assembled within EnteroBase [1]. It was intended to provide a venue for bioinformatic approaches, and lead to insights on how to analyse and learn from such enormous numbers of pathogen genomes. However, the schedule overlapped with the COVID-19 pandemic, and the meeting was postponed to avoid the possibility of a super-spreader event at a live meeting of 100s of individuals. The further course of the COVID-19 pandemic highlighted both the tremendous analytical challenges that result from large-scale pathogen sequencing as well as the novel opportunities that they can provide.

The postponed discussion meeting ultimately took place in September 2021 as an online event. During the intervening 18 months, the world of pathogen sequencing and analytics changed dramatically—over 3.9 million genomic sequences of SARS-CoV-2 virus became publicly available [2], pushing existing phylogenetic and visualization tools to their absolute limits and sparking the creation of several new solutions. Routine sequencing of bacterial pathogens also continued, and became even more common in some settings owing to increased accessibility of sequencing in clinical and public health settings. By June 2022, more than 11.5 million SARS-CoV-2 genome sequences had been deposited in the GISAID database (https://www.gisaid.org/), and there were over 430 000 *Salmonella* sequences in more than 21 000 single nucleotide polymorphism (SNP) clusters within the NCBI Pathogen Detection Portal (https://www.ncbi.nlm.nih.gov/pathogens/about/). That portal and EnteroBase also contain large numbers of assembled genomes of bacterial pathogens from multiple other genera. The era of big-data pathogen genomics has now well and truly arrived.

This special issue highlights some of the new ideas and methodological approaches that were contributed by speakers at the September 2021 meeting. Each article brings a fresh perspective to the topic of understanding genomic population structures of microbial pathogens, including scaling up phylogenetic analyses; developing tree-free alternatives to phylogenetics; understanding the influence of horizontal gene transfer in bacteria; and using genomics to explore adaptation at different scales.

## 2. Phylogenetic approaches to understanding population structure

Fisher *et al*. [3] provide a brief overview of Bayesian phylogenetics for a broad audience. This approach has largely been limited to medium-sized datasets until recently because it depends on computationally intensive Markov chain Monte Carlo (MCMC) sampling of all plausible evolutionary alternatives. Recent methodological approaches are described that can improve the speed of likelihood calculations, reduce the time needed for MCMC burn-in, and generate improved MCMC estimates. Burn-in times were reduced from 6 h for a naive analysis on 720 SARS-COV-2 genomes of which 588 had been previously analysed, to less than 1 min, by adding the 132 new genomes to the pre-existing 588-tip tree. MCMC proposals for phenotypic evolution in a 1536-tip human immunodeficiency virus (HIV) tree were generated 1000 times more quickly during the main run by parallel computing based on Hamiltonian MCMC in comparison to adaptive MCMC (uMH). Similarly, the age of a SARS-COV-2 clade within a 1000-tip genome tree was calculated in as little as 7% of the runtime needed by uMH. The article concludes with a discussion of alternative methods that do not rely on MCMC for estimating the posterior likelihood.

Didelot & Parkhill [4] describe the advantages of using a step-by-step approach for interpreting bacterial epidemiology from genomic sequences. Their approach consists of: generating sequence alignments, identifying recombinant imports with a distinct phylogenetic history, calculating an unrooted phylogeny, and elucidating a dated tree and population structures. These steps then facilitate elucidating the transmission chains that are of interest to epidemiologists. This article compares the merits of alternative programmes for each step, and makes a strong argument that a multi-step approach is safer than relying on one massive model that tries to estimate all the different parameters at once. The conclusions are illustrated with an example of such calculations with 529 assembled genomes of *Staphylococcus aureus* ST239 from global sources, including details of the time needed to perform each of the steps on a laptop computer.

Zaharias & Warnow [5] provide a broad overview of new methods that can generate highly accurate maximum likelihood (ML) trees from multiple sequence alignments containing 100 000 s of gene or genomic sequences. Many of these methods rely on divide-and-conquer techniques that can handle phylogenetic heterogeneity resulting from incomplete lineage sorting and/or gene duplication or loss. Still other methods can add sequences into large, pre-existing gene or species trees. The different methods that are available for each stage of the process are evaluated for speed and accuracy, and this manuscript will serve as a highly useful source of information on how to most accurately and most rapidly create gene and species trees from very large datasets of genomic data.

Phylogenetic trees are used to calculate evolutionary processes and estimate the historical dates of genetic splits. However, they are also used routinely to infer population structures and epidemiological events although the theoretical basis for such inferences has not been definitively clarified. In their publication, Hayati *et al.* [6] use novel deep learning approaches to distinguish nine tree shapes that appear repeatedly among genome sequences of multiple species containing bacterial pathogens. Several sub-tree shapes could be assigned visually to the so-called ‘comet’, ‘star’ or ‘barbell’ types, which may be relevant for epidemiological purposes. The ‘comet’ type contains a few ancestral genomes on long branches whose isolates predated the expansion of a cluster of closely related isolates. The ‘star’ type includes multiple discrete branches of comparable length, and might be associated with repeated homologous recombination or rapid diversification without much selection. The ‘barbell’ shape contains two discrete sub-populations with a long internal branch separating them. However, several other shapes were also distinguished by deep learning, each encompassing distinct subpopulations, and represent a population structure which warrants further investigation. A variety of statistical tools demonstrated only limited association with geographical range, date of isolation or other metadata, illustrating the problems which are posed for detailed epidemiological interpretations by genomic sequences without extensive associated metadata.

## 3. Alternatives to phylogenetics

Achtman *et al*. [7] present a monograph on the suitability of HierCC (hierarchical clustering of core genome multi-locus sequence typing data (cgMLST)) within EnteroBase, for the identification of species and sub-species in six bacterial genera: *Salmonella, Escherichia/Shigella*, *Clostridioides*, *Yersinia*, *Vibrio* and *Streptococcus.* For each genus, a large representative dataset of assembled genomes is identified. ML trees that were calculated based on core SNPs and on the presence/absence of accessory genes yielded similar branching topologies. The branch structures of these trees were then used as the ‘ground truth’ for taxonomic designations in comparisons with published designations, clusters based on 95% average nucleotide identity (95% ANI) and HierCC clusters. HierCC was found to be more sensitive, accurate and consistent than classical taxonomy or ANI for five of the genera, and was used to guide numerous manual taxonomic changes that are now stored in EnteroBase, including the definition of novel species and sub-species, deletion of scores of incorrect species designations as well as upgrading/downgrading the taxonomic levels of other species. Exceptionally, it was not possible to identify a DNA-based metric that was consistent with the taxonomic designations of *Streptococcus* because multiple ANI and HierCC clusters at the species/sub-species level were identified in several taxonomic species. The manuscript also describes the correlations between HierCC and populations/lineages in *Salmonella* and *Escherichia/Shigella*. HierCC is concluded to represent a worthy replacement for legacy MLST within these genera. Lineages within *Salmonella* are remarkably uniform in O serogroup, whereas O serogroup is highly variable even within sequence type (ST) complexes within *Escherichia*. The manuscript also includes comparisons of EnteroBase HierCC and Mandrake (introduced by Lees *et al.* [8] in this special issue) on a large number of public genomes of *Streptococcus pneumoniae*. The two methods were highly consistent.

Lees *et al*. [8] describe Mandrake, a new approach that can cluster 600 000 bacterial genomes or one million SARS-COV-2 genomes within a few hours. It is an efficient implementation of dimensional reduction that can be run in parallel mode on a GPU processor, and provides comparable or better clustering of simulated population structure of bacterial genomes than other slower methods including principal components analysis, t-distributed stochastic neighbour embedding and uniform manifold approximation and projection. Mandrake was also applied to a gene presence/absence matrix of 20 047 genomes of *S. pneumoniae* and yielded very similar clustering to either PopPunk clusters based on core plus accessory genes or HC160 clusters according to EnteroBase HierCC [9]. Similar results were obtained with *Salmonella,* where Mandrake clusters correlated with a somewhat finer clustering than HC900 in HierCC. Similarly, Mandrake clustered *Listeria monocytogenes* and *Mycobacterium tuberculosis* according to their major lineages. Similar clustering was also observed with 600 000 bacterial genomes of diverse taxa, which were largely clustered into species-specific lineages, and one million SARS-COV-2 genomes in which the major variants of concern were assigned to variant-specific clusters.

## 4. Influence of mobile genetic elements on population structure and inference

In their article, Haudiquet *et al.* [10] present an overview of how mobile genetic elements (MGEs) drive horizontal gene transfer in microbial populations. It paints a picture of bacterial genomes as playgrounds for MGEs engaged in evolutionary war-games, which result in gene flow that is a rich provider of novel functions to microbial genomes but is largely out of the control of the recipient cells. Haudiquet, Rocha and colleagues discuss mechanisms by which MGEs control the timing of their own transfer. For example, SOS responses of the host bacteria are used as cues to ‘jump ship’ to neighbouring cells, and quorum-sensing systems are used to detect the availability of suitable new hosts in the vicinity. The manuscript then considers bacterial host factors that control or constrain MGEs, including variation in the cell envelope and associated structures as well as restriction-modification systems, and how their interplay with MGE create mobile gene turnover and shape the evolution of the pan-genome. Finally, the authors reflect on the need for quantitative studies which can measure how these complex mechanisms contribute to adaptive evolution of bacteria.

Quantitative data on horizontal gene transfer (HGT) can be gathered by investigating naturally occurring gene and plasmid transfer events. This is of increasing interest in the field of infection control genomics, because the transfer between strains of plasmids and antimicrobial resistance (AMR) genes is now well documented as playing a pivotal role in the emergence and persistence of hospital-associated outbreaks of drug resistant infections [11]. In their article, Huisman *et al.* [12] consider how the choice of sequencing and assembly methods can impact inferences of HGT of plasmids and AMR genes. They use a set of 24 *Escherichia coli* isolates, sequenced with the three currently most-popular technology platforms (Illumina, Oxford Nanopore and PacBio), and use cgMLST gene alignments for phylogenetic inference of chromosomal trees with BEAST2. The results show strong topological similarity for Illumina, PacBio and Illumina/Nanopore hybrid genomes, regardless of sequencing platform or assembly method. However, assemblies based exclusively on Oxford Nanopore sequences resulted in erroneous tree topologies because they were prone to base-call errors that interfered with cgMLST gene calling. Inference and comparisons of plasmid trees are challenging because plasmid sequences can be fragmented across contigs. Huisman *et al.* [12] use an interesting approach of aligning all genes that are annotated on the same contig as a shared plasmid replicon marker. This approach revealed substantial variation in the plasmid trees generated using different sequencing and assembly methods. However, downstream inferences of plasmid transfer between strains, and of the transfer of plasmid-encoded AMR genes, were quite robust to methodological choices. These findings are encouraging for the burgeoning field of plasmid genomics and transmission tracking [13], the importance of which is increasingly recognized in public health genomics and infection control.

## 5. Understanding adaptation at different scales

Fine-scale changes within an individual's microbiome, including strain-level adaptations, may contribute to a person's health and wellbeing. In her review article, Lieberman [14] explores the potential for tracking adaptive evolution within individual microbiomes at short timescales. Understanding selection pressures exerted upon microbial genes and genomic loci within an individual microbiome has the potential to inform the design of rational probiotic therapies and contribute to a deeper understanding of the fine-scale forces exerted upon populations within a therapeutic regime. Current technical limitations to whole-genome-scale investigation of selective pressures on microbes are discussed, along with potential solutions. For example, culture-based approaches for exploring sub-lineage dynamics within multi-strain scenarios are successfully uncovering phylogenetic details of lineages within microbiomes. Lieberman [14] notes that the extrapolation of insights from haploid eukaryotes to asexual bacteria can lead to underestimating within-host recombination rates in prokaryotes and the resulting adaptive evolution. Lieberman [14] maps out the potential landscape for adaptive change: given a conservative rate of 10^−10^ mutations per cell division, a population of 10^10^ cells within a person's gut could explore every single point mutation across the genome each generation. She describes challenges with detection, limitations of current statistical methods, and provides a strong case for using parallel evolution as an indicator of recent within-host adaptation. This approach is illustrated with examples from the bacterial literature. She also compares the use of different operational units (nucleotides, codons, genes, operons or pathways) at the genomic scale and at geographical scales (e.g. between people versus intra-person), and argues that understanding the factors which determine adaptive potential will focus efforts to link in-person mutations to health and disease.

Models of metabolism within a genomic scale can be used to characterize both pan-genome and core genomic capabilities in model organisms. However, population level dynamics require phylogeny-based approaches. Here, Monk [15] constructs genome-scale metabolic models for 222 strains of *Escherichia* spanning the breadth of the core-genome diversity represented within Enterobase. Differences within and between species and strains are described and used to demarcate core and pan metabolic capabilities within *Escherichia* along with the calculation of growth phenotypes on over 400 nutrients. This analysis paves a path for determining common metabolic capabilities at the genus level via *in silico* inference. Within the context of pan-*Escherichia* metabolism, Monk also describes multiple niche adaptations in metabolic capabilities. Finally, he stresses the importance of the development of a highly curated community resource for elucidating genotype to phenotype relationships, and which could provide contextual data for the rapid construction of strain-specific models for newly sequenced isolates.

## 6. Other topics covered

The ‘Genomic population structures of microbial pathogens' meeting (https://royalsociety.org/science-events-and-lectures/2021/09/microbial-pathogens/) included several other noteworthy presentations that are not covered by articles in this collection. Zamin Iqbal spoke about a key conceptual problem in bacterial genomic analysis: how to capture and interpret information on population structure and function that is embedded in the accessory genome. One solution, proposed by Iqbal and colleagues and implemented in their tool Pandora [16], is to build a pangenome graph to represent a population of genomes, and use this as a reference against which to call single nucleotide and gene content variation. David Aanensen spoke about Pathogenwatch, an online platform for rapid local and international genomic epidemiology, which is supporting genomic surveillance and outbreak investigations for a range of pathogens including *Neisseria gonorrhoeae*, *Klebsiella pneumoniae* and *Salmonella* enterica serovar Typhi (typhoid fever). Kat Holt continued this theme by discussing 20 years of typhoid genomics, culminating in recent efforts by the Global Typhoid Genomics Consortium to aggregate Typhi genomics data [17], make it accessible to non-genomics audiences via the online dashboard TyphiNET; and facilitate widespread use of genomic data to inform public health policy including building the case for local rollout of new typhoid vaccines. Pybus summarized insights into the SARS-CoV-2 pandemic and other outbreaks from large scale phylogenetic analysis [18]; and Emma Hodcroft presented the CoVariants dashboard, which allows interactive exploration of SARS-COV-2 variants of concern. Finally, Christophe Fraser spoke about harnessing within- and between-host pathogen population genetics, using the approach implemented in Phyloscanner [19], to gain insights into the epidemiology, evolution and pathogenicity of diverse pathogens ranging from methicillin resistant *Staphylococcus aureus* (MRSA) [20] to SARS-COV-2 and HIV [21].

## 7. Final words

In 2006, a Royal Society meeting on ‘Species and speciation in microorganisms’ was coupled with a special issue of *Phil. Trans. R. Soc. B.* That meeting was organized by Matthew Fisher, Brian Spratt and James Staley [22], and had an enormous impact on the thinking of microbial population geneticists. Memories of that meeting triggered the editors of this introduction in conceiving of a similar meeting and special issue dealing with solutions and insights on the problems and potential of Big Data in genomic sequences of pathogenic microbes. That was 4–5 years ago. It is therefore with an enormous sigh of relief that we perceive the publication of this special issue. It would have been wonderful to have had a live meeting at which we could discuss these topics in detail with other colleagues that we have not seen in years. It would have been a superb opportunity to introduce insights on this topic to a broad audience. However, all three editors are proud to have found the courage to cancel the live meeting two weeks prior to its scheduled date at the end of March 2020, and very grateful to the Royal Society for the grace with which they agreed to this cancellation. We heartily thank the staff at the Royal Society who so successfully managed the virtual meeting in 2021, and Helen Eaton, the Editor at *Phil. Trans. R. Soc. B* who demonstrated such incredible patience with our delays in providing accepted manuscripts for this special issue. However, our greatest thanks go to the authors of the individual jewels in this special issue for providing such lucid explanations of difficult topics.

## Data Availability

This article has no additional data.
